# DNA Microarray Analysis on the Genes Differentially Expressed in the Liver of the Pufferfish, *Takifugu rubripes*, Following an Intramuscular Administration of Tetrodotoxin

**DOI:** 10.3390/microarrays3040226

**Published:** 2014-10-27

**Authors:** Takuya Matsumoto, Holger Feroudj, Ryosuke Kikuchi, Yuriko Kawana, Hidehiro Kondo, Ikuo Hirono, Toshiaki Mochizuki, Yuji Nagashima, Gen Kaneko, Hideki Ushio, Masaaki Kodama, Shugo Watabe

**Affiliations:** 1 Department of Aquatic Bioscience, Graduate School of Agricultural and Life Sciences, The University of Tokyo, Bunkyo, Tokyo 113-8657, Japan; E-Mails: takuya62@pu-hiroshima.ac.jp (T.M.); Holger.Feroudj@gmail.com (H.F.); ryosuke-1129@hotmail.co.jp (R.K.); agkaneko@mail.ecc.u-tokyo.ac.jp (G.K.); aushio@mail.ecc.u-tokyo.ac.jp (H.U.); akodama1943@yahoo.co.jp (M.K.); 2 Laboratory of Genome Science, Graduate School of Marine Science and Technology, Tokyo University of Marine Science and Technology, Minato, Tokyo 108-8477, Japan; E-Mails: qqq69dododo@yahoo.co.jp (Y.K.); h-kondo@kaiyodai.ac.jp (H.K.); hirono@kaiyodai.ac.jp (I.H.); 3 Kawaku Company Limited, Shimonoseki, Yamaguchi 750-0093, Japan; E-Mail: mochizuki@kawaku.com; 4 Department of Food Science and Technology, Graduate School of Marine Science and Technology, Tokyo University of Marine Science and Technology, Minato, Tokyo 108-8477, Japan; E-Mail: yujicd@kaiyodai.ac.jp; 5 School of Marine Biosciences, Kitasato University, Minami, Sagamihara, Kanagawa 252-0373, Japan

**Keywords:** tetrodotoxin, pufferfish *Takifugu rubripes*, microarray analysis, gene expression, intramuscular administration, accumulation, toxification

## Abstract

Pufferfish accumulate tetrodotoxin (TTX) mainly in the liver and ovary. This study aims at investigating the effect of TTX accumulation in the liver of cultured specimens of torafugu *Takifugu rubripes* on the hepatic gene expression by microarray analysis on Day 5 after the intramuscular administration of 0.25 mg TTX/kg body weight into the caudal muscle. TTX was detected in the liver, skin and ovary in the TTX-administered individuals. The total amount of TTX accumulated in the body was 67 ± 8% of the administered dose on Day 5. Compared with the buffer-administered control group, a total of 59 genes were significantly upregulated more than two-fold in the TTX-administered group, including those encoding chymotrypsin-like elastase family member 2A, transmembrane protein 168 and Rho GTP-activating protein 29. In contrast, a total of 427 genes were downregulated by TTX administration, including those encoding elongation factor G2, R-spondin-3, nuclear receptor activator 2 and fatty acyl-CoA hydrolase precursor. In conclusion, our results demonstrate that the intramuscular administration of TTX changes the expression of hepatic genes involved in various signaling pathways.

## 1. Introduction

Tetrodotoxin (TTX) is a potent neurotoxin, which binds to voltage-gated sodium channels with a very high affinity, and it is generally accepted that TTX is accumulated at high levels in specific tissues, such as the liver, ovary and skin, of *Takifugu pufferfish* [1,2]. The hypothesis that pufferfish themselves are unable to synthesize TTX is now widely accepted, which is mainly supported by the fact that cultured specimens are non-toxic, but become toxic by feeding TTX-containing artificial diets [3,4,5,6,7]. TTX was also found in various wild marine animals, such as worms, annelids, snails, starfish and crabs [8]. In addition, TTX-producing marine bacteria were isolated from pufferfish, xanthid crab and red calcareous alga, as well as from marine environments [9,10,11,12,13,14]. These results support that TTX is an exogenous substance for TTX-bearing organisms, and the toxification occurs via the food chain, bacterial parasitism or symbiosis [15,16].

We investigated the *in vivo* pharmacokinetics of TTX in cultured specimens of torafugu *T. rubripes* given a single intravenous and gastrointestinal administration [17,18]. TTX was well introduced into the systemic circulation from the gastrointestinal tract by a saturable mechanism and rapidly taken up into the liver. In addition, we developed tissue models of *in vitro* accumulation/uptake of TTX in the liver, revealing the involvement of the carrier-mediated transport system in the TTX uptake mechanism of torafugu *T. rubripes* [19,20,21]. These results strongly indicate that pufferfish have a special function to actively accumulate TTX in the liver at high concentrations.

We previously investigated the genes related to the accumulation of TTX in the liver by comparing mRNA expression patterns in the wild marine pufferfish, *T. chrysops* and *T. niphobles*, which have different concentrations of TTX in the liver using mRNA arbitrarily-primed (RAP) RT-PCR [22]. Briefly, RAP RT-PCR provided a 383-bp cDNA fragment, and its transcripts were higher in toxicity than non-toxic pufferfish liver. Its deduced amino acid sequence was similar to those of fibrinogen-like proteins reported for other vertebrates. The cDNA fragment of 383 bp was composed of at least three fibrinogen-like protein (flp) genes (flps), flp-1, flp-2 and flp-3, in the liver of *T. chrysops* and *T. niphobles* containing high concentrations of TTX, and the relative mRNA levels of these genes showed a linear correlation with TTX levels in the liver of the two species. The gene encoding flp-1 in the liver of *T. niphobles* located in scaffold 628 of the Fugu Genome Database, and the amino acid sequence in a C-terminal region of flp-3 in *T. chrysops* liver was homologous to hepcidin precursors of the spotted green pufferfish, *Tetraodon nigroviridis*, European sea bass *Dicentrarchus labrax*, mouse and human. In addition, we also examined the hepatic gene expression profile in cultured torafugu by suppression subtractive hybridization (SSH) at 12 h after the intramuscular administration of 0.50 mg TTX/kg body weight into the caudal muscle [23]. The intramuscular administration of TTX increased the transcripts encoding acute-phase response proteins, such as hepcidin, complement C3, serotransferrin, apolipoprotein A-1 and high temperature adaptation protein Wap65-2 in the liver at 12 h after administration. Very recently, we performed DNA microarray analysis with total RNAs from toxic and non-toxic wild pufferfish [24]. The mRNA levels of 1,108 transcripts were more than two-fold higher in toxic than in nontoxic specimens, and the expression levels of nine genes were upregulated more than 10-fold in toxic ones. It was noted that proteins encoded by these genes are related to vitamin D metabolism and immunity. It is unclear, however, whether the transcripts of these genes are involved in TTX disposition and how they function in pufferfish. Thus, the biological and physiological significance of TTX in pufferfish remains unknown.

In this study, we performed DNA microarray analysis on the liver of marine pufferfish *T. rubripes* at five days after the intramuscular administration of 0.25 mg TTX/kg body weight into the caudal muscle to investigate the effect of TTX accumulation into the liver on the hepatic gene expression and to identify the genes possibly related to TTX accumulation in the liver. This study would answer questions beyond just the transcriptomic changes that may help drive TTX accumulation in the liver and also contribute to better understanding how the transcriptomic response can limit the toxicity of TTX to pufferfish.

## 2. Experimental Section

### 2.1. Materials

Experimental marine pufferfish *T. rubripes* specimens (18 months old, approximately 1 kg body weight), cultured by the flow-through aquaculture system that efficiently utilized the underground seawater from the Kanmon Tunnel at the Aquaculture Station, Kawaku Co., Ltd. in Shimonoseki, Yamaguchi Prefecture, Japan, were used in the present study. The temperature of the seawater was constant at around 20 °C throughout the year, and fish were fed arbitrarily with commercial diets. TTX used in the administration study was purified from the ovary of wild pufferfish *T. rubripes* collected at the coastal area of the Genkai-nada Sea in Japan by a combination of ultrafiltration and a series of column chromatographic separations, as reported previously [21]. Crystalline TTX (Wako Pure Chemicals Industries, Osaka, Japan) was used as a standard for the liquid chromatography-fluorescence detection (LC-FLD) analysis. All other reagents were of analytical grade.

### 2.2. TTX Administration and Sample Preparation

The administration experiments were carried out at Kawaku Aquaculture Station in September, 2010. TTX was dissolved in modified Hank’s balanced salt solution buffer (160 mM NaCl, 5.4 mM KCl, 0.34 mM Na_2_HPO_4_, 0.44 mM KH_2_PO_4_, 10 mM 4-(2-hydroxyethyl)-1-piperazineethanesulfonic acid, adjusted to pH 7.4 with NaOH solution), and five pufferfish specimens (0.99 ± 0.06 kg body weight) received an intramuscular injection of 0.25 mg TTX/500 µL/kg body weight into the caudal muscle and were maintained in a 1000-L circular culture tank using the flow-through aquaculture system for 5 days at 20 °C (the TTX-administration group, city, state, country). On Day 5 after administration, these fish were removed from the circular culture tank, and their tissues were dissected. The liver was cut into 5-mm pieces, immediately stored in RNAlater solution (Applied Biosystems, Foster City, CA, USA) and stored at −80 °C until use. For the control group, five pufferfish specimens (1.02 ± 0.06 kg body weight) were given an intramuscular injection of the buffer (500 µL/kg body weight) that did not contain TTX, and the liver samples were prepared as described above. The remaining liver samples and other tissues (ovary, skin, and muscle) were stored at −20 °C for TTX determination by LC-FLD.

### 2.3. TTX Extraction and Quantification

TTX was extracted from the tissue samples with 0.1% acetic acid by heating in a boiling water bath for 10 min after ultrasonication for 1 min according to the standard assay procedures for TTX [25]. TTX quantification was performed by LC-FLD analysis according to the methods of Nagashima *et al.* [26] and Shoji *et al.* [27] with some modifications. Briefly, the analytical column was a Wakopak Navi C30-5 (4.6 mm i.d. × 250 mm, 5 mm particle size, Wako Pure Chemical Industries, Osaka, Japan) and maintained at 25 °C. The mobile phase consisted of 5 mM sodium heptanesulfonate in 10 mM ammonium formate (pH 5.0) containing 1 vol% acetonitrile and was eluted at a flow rate of 1.0 mL/min. The eluates were heated at 105 °C with 4 N NaOH (flow rate 1.0 mL/min) in a Teflon tubing (0.5 mm i.d. × 20 m). The reaction products were cooled by flowing through the stainless tube (0.46 mm i.d. × 0.3 m) kept in ice-cold water and detected by an FP-2020 fluorescence detector (JASCO, Tokyo, Japan) with excitation at 365 nm and emission at 510 nm.

### 2.4. RNA Extraction for Microarray Analysis

Total RNAs were extracted from the liver samples of each group using the RNeasy Lipid Tissue Mini Kit (Qiagen, Hilden, Germany), as described in the manufacturer’s instructions. Total RNA concentrations were measured using a NanoPhotometer (IMPLEN, Munich, Germany), and the quality of total RNA was analyzed by agarose gel electrophoresis.

### 2.5. Preparation of Fluorescently-Labeled cRNA and Microarray Analysis

A custom 44k oligonucleotide microarray was designed using the Agilent eArray application (Agilent Technologies, Santa Clara, CA, USA) [28] based on the predicted cDNA data (FUGU version 4) of the genome assembly [29]. A 700-ng aliquot of total RNAs extracted from the liver sample each of TTX-administered and control groups was mixed with One-Color Spike-Mix (Agilent Technologies), reverse-transcribed and labeled with Cy3 using the Quick Amp Labeling Kit (Agilent Technologies). Cy3-labeled cRNA was purified using the RNeasy Mini Kit (Qiagen) and fragmented using the Gene Expression Hybridization Kit (Agilent Technologies). The samples were mixed with an equal volume of hybridization buffer and transferred on the microarray slide glass, which was subsequently incubated at 65 °C for 17 h. After hybridization, the microarray glass slides were washed with gene expression wash Buffer 1 at room temperature for 1 min and rinsed with gene expression wash Buffer 2 at 37 °C for 1 min. The slide glasses were then dried with nitrogen gas and scanned immediately using a GenePix4000B scanner (Axon Instruments, Foster City, CA, USA). The scanning image files were converted into expression data using Feature extraction software version 10.7.3 (Agilent Technologies) [30]. Microarray data analysis was performed using GeneSpring GX software version 11.0 (Agilent Technologies) [31]. The raw expression values were normalized, and gene expression ratios were calculated by normalizing the TTX-administered *versus* control group. Differentially expressed genes in the TTX-administered group were selected based on a >2.0-fold change.

### 2.6. Quantitative Real-Time PCR

The differential expression of chymotrypsin-like elastase family member 2A (cela2a), which was the highest upregulated gene in the TTX-administered group, was further validated by quantitative real-time PCR. Briefly, total RNAs were extracted from the liver samples by the above protocol and treated with DNase I (Invitrogen, Carlsbad, CA, USA). First-strand cDNAs were constructed using oligo-dT20 primers and SuperscriptTM III reverse transcriptase (Invitrogen), as described in the manufacturer’s instructions. Real-time PCR was performed in a 20-µL reaction mixture containing 2 µL of cDNA (1:20 dilution), 10 µL of SYBR Premix Ex Taq II (Takara Bio, Otsu, Japan), 0.4 µL of ROX reference dye (Takara Bio), 0.8 µL of 10 µM gene-specific forward primer and 0.8 µL of 10 µM gene-specific reverse primer on an ABI7300 Real-Time PCR System (Applied Biosystems). Reactions were as follows: 95 °C for 30 s; then 40 cycles of 95 °C for 5 s and 60 °C for 31 s. The relative fold change of the cela2a mRNA expression level was determined by the comparative delta threshold cycle (ΔCt) method for relative quantification based on the beta actin 1 mRNA (Accession Number U37499.1) expression level. The gene-specific primers were designed using Primer Express software version 3.0 (Applied Biosystems) [32], and the sequences are as follows: 5'-CTCTTCCAGCCATCCTTCCTT-3' (forward) and 5'-GACGTCGCACTTCATGATGCT-3' (reverse) for beta actin 1 (Accession No. U37499.1) and 5'-GGCACCACACCTTCAATCCT-3' (forward) and 5'-GGCTGGGAACAGATGGA ATG-3' (reverse) for cela2a (Ensemble FUGU ID, ENSTRUT00000045544).

### 2.7. Data Analysis and Statistics

Data from the quantitative real-time PCR are expressed as the mean ± standard error (SE), and the Student’s *t*-test was used to analyze the significance of differences among the means at the level of *p* < 0.05.

## 3. Results

### 3.1. TTX Determination

TTX in the tissues of pufferfish *T. rubripes* on Day 5 after administration was analyzed by LC-FLD. TTX was detected only in the tissues from the TTX-administered group, but not from the control group (<0.15 µg TTX/g tissue). The concentration and total amounts of TTX in the tissues are summarized in Table 1. The concentration was highest in the liver followed by the skin and ovary, whereas TTX in the muscle was not at the detectable level. The ratio of the total amount of TTX accumulated to that administered was quite high (about 70%).

**Table 1:** Tetrodotoxin (TTX) concentrations and contents in the tissues of *Takifugu rubripes* specimens in the TTX-administered group.

No.	Sex	Body weight (kg)	Dose (µg)	TTX concentration (µg/g)				Total amount (µg)	Accumulation (% of dose)
				Liver	Skin	Ovary	Muscle		
1	F	1.14	285	0.48	0.30	5.43	N.D.^1^	145	51
2	F	0.84	210	0.68	0.97	5.30	N.D.	162	77
3	F	1.00	250	1.12	0.62	4.97	N.D.	206	82
4	F	1.10	275	0.54	0.33	4.51	N.D.	124	45
5	M	0.86	215	1.35	0.79	N.D. ^1^	N.D.	176	82
Mean ± SE		0.99 ± 0.15	247 ± 15	0.84 ± 0.17	0.60 ± 0.13	5.05 ± 0.21	N.D.	163 ± 14	67 ± 8

^1^ N.D.: not detected (<0.15 µg TTX/g tissue).

### 3.2. Gene Expression Analysis

**Figure 1:**
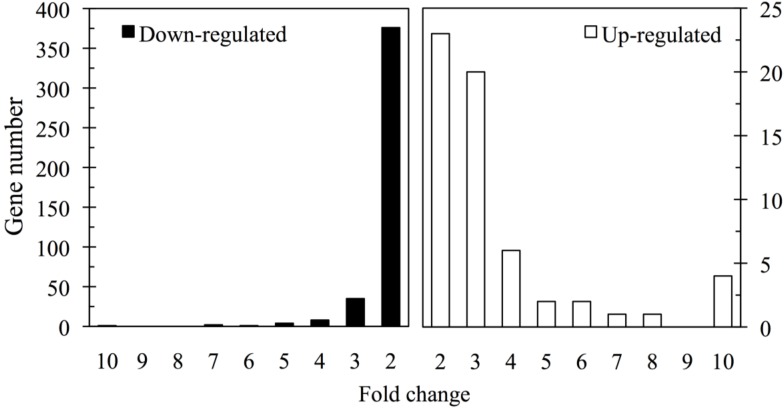
Fold change analysis of 486 genes differentially expressed in the liver of *Takifugu rubripes*. The histogram shows the fold change values for 427 downregulated genes (**left**) and 59 upregulated genes (**right**) in the liver of *T. rubripes* on Day 5 after the intramuscular administration of TTX.

**Figure 2:**
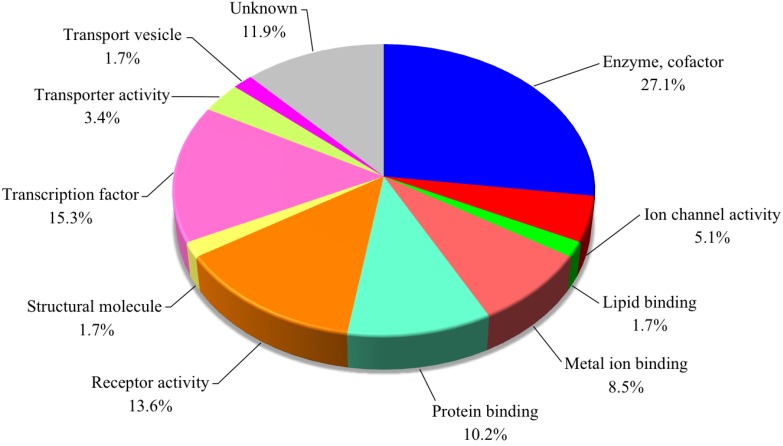
Functional classification of 59 genes significantly upregulated in the liver of *Takifugu rubripes* in the TTX-administered group.

To identify the differentially expressed genes between the TTX-administered pufferfish *T. rubripes* liver and control, the cDNA microarray analysis was performed using the Agilent eArray platform. A total of 59 genes were found to be upregulated more than two-fold with a *p*-value <0.05 in the TTX-administered group, as shown in Figure 1. The genes upregulated three-fold and more were extracted and are listed in Table 2. The highest upregulated gene was chymotrypsin-like elastase family 2A, and its fold change (FC) value was 37.6. The upregulated genes were assigned to have major molecular functions of the putative translated proteins based on their gene ontology information and the Gene Ontology Database [33] As shown in Figure 2, genes involved in enzyme and cofactors (metabolism) and transcription accounted for 27.1% and 15.3%, respectively, whereas those involved in receptor activity, protein binding and metal ion binding accounted for 13.6%, 10.2% and 8.5%, respectively.

**Table 2:** List of genes upregulated in the liver of the TTX-administered pufferfish *Takifugu rubripes* group compared to those in the buffer-administered control group (FC ^1^ >3.0).

Ensemble ID	Gene name	Predicted description	Functional classification	FC
ENSTRUT00000045544	Cela2a	Chymotrypsin-like elastase family member 2A	Enzyme, cofactor	37.6
ENSTRUT00000041722	Tmem168	Transmembrane protein 168	Transport vesicle	20.0
ENSTRUT00000006023	Arhgap29	Rho GTPase-activating protein 29	Enzyme, cofactor	12.1
ENSTRUT00000034782	Kcnma1	*T. rubripes* calcium channel alpha-1 subunit homolog (AF026198.1)	Ion channel activity	10.4
ENSTRUT00000004724	Chrna9	*T. rubripes* nicotinic acetylcholine receptor alpha 9d subunit (AY299471.1)	Ion channel activity	8.1
ENSTRUT00000038610	Csmd1	CUB and sushi domain-containing protein 1	Protein binding	7.0
ENSTRUT00000036792	Serinc4	Serine incorporator 4	Transporter activity	6.8
ENSTRUT00000026094	C14orf135	Pecanex-like protein C14orf135	Unknown	6.6
ENSTRUT00000046072	Lrp2	Low density lipoprotein receptor-related protein 2	Receptor activity	5.4
ENSTRUT00000046718	Col27a1	Collagen alpha-1(XXVII) chain A-like	Structural molecule	5.2
ENSTRUT00000000397	Klhl32	Kelch-like protein 32	Protein binding	4.5
ENSTRUT00000000173	Elk4	ELK4, ETS-domain protein	Transcription factor	4.3
ENSTRUT00000025902	Zbtb45	Zinc finger and BTB domain-containing protein 45	Transcription factor	4.3
ENSTRUT00000020252	Spsb1	SPRY domain-containing SOCS box protein 1	Receptor activity	4.3
ENSTRUT00000032342	Dysf	Dysferlin	Lipid binding	4.1
ENSTRUT00000003310	Scn2b	Sodium channel beta-2 subunit	Ion channel activity	4.0
ENSTRUT00000011167	Loxl2	Lysyl oxidase homolog 2	Enzyme, cofactor	3.9
ENSTRUT00000017057	Dmbx1	Diencephalon/mesencephalon homeobox protein 1	Transcription factor	3.9
ENSTRUT00000023088	Clk2	Dual specificity protein kinase CLK2	Enzyme, cofactor	3.9
ENSTRUT00000045827	Myo3b	Myosin-IIIb	Enzyme, cofactor	3.8
ENSTRUT00000025143	Megf11	Multiple epidermal growth factor-like domains protein 11	Metal ion binding	3.7
ENSTRUT00000003539	Slc44a1	Choline transporter-like protein 1	Transporter activity	3.7
ENSTRUT00000017798	Cc4	Carbonic anhydrase 4	Enzyme, cofactor	3.5
ENSTRUT00000006962	OR4563-2	*T. rubripes* odorant receptor (DQ306241.1)	Receptor activity	3.5
ENSTRUT00000024316	Pitpnm2	Membrane-associated phosphatidylinositol transfer protein 2	Metal ion binding	3.4
ENSTRUT00000030952	Ptafr	Platelet-activating factor receptor	Receptor activity	3.4
ENSTRUT00000038903	Hepacam2	HEPACAM family member 2	Unknown	3.4
ENSTRUT00000043658	Gpr22	Probable G-protein coupled receptor 22	Receptor activity	3.3
ENSTRUT00000031306	Ptpn2	Tyrosine-protein phosphatase non-receptor type 2	Enzyme, cofactor	3.3
ENSTRUT00000019629	Eps15l1	Epidermal growth factor receptor substrate 15-like 1	Receptor activity	3.2
ENSTRUT00000036590	Zdhhc8	Membrane-associated DHHC8 zinc finger protein (NM_001078596.1)	Enzyme, cofactor	3.2
ENSTRUT00000031045	Rheb	GTP-binding protein Rheb	Enzyme, cofactor	3.2
ENSTRUT00000047583	Agap2	Arf GAP with GTPase domain, ankyrin repeat and PH domain 2	Enzyme, cofactor	3.0
ENSTRUT00000017824	Ppargc1a	Peroxisome proliferator activated receptor gamma coactivator 1 alpha (DQ157766.1)	Receptor activity	3.0
ENSTRUT00000027961	Pfkp	6-phosphofructokinase type C	Enzyme, cofactor	3.0
ENSTRUT00000029743	E2f2	Transcription factor E2F2	Unknown	3.0

^1^ FC is the average fold change of the TTX-administered (n = 5) compared to the buffer-administered control group (n = 5).

**Figure 3:**
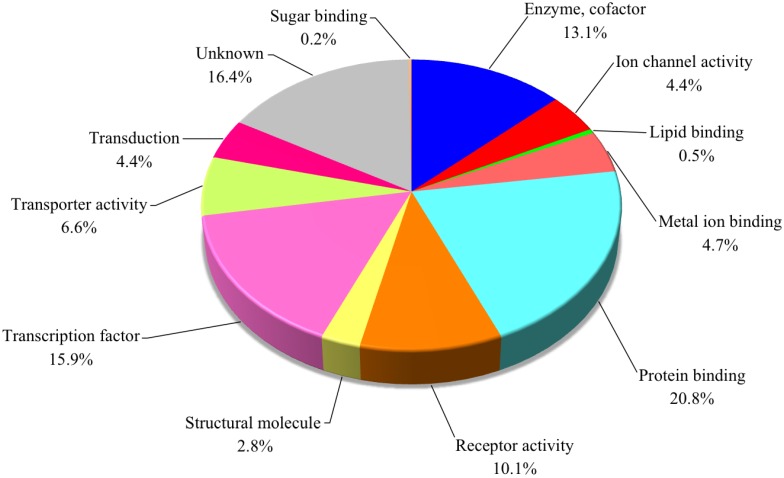
Functional classification of 427 genes significantly downregulated in the liver of *Takifugu rubripes* in the TTX-administered group.

In contrast, a total of 427 genes were found to be downregulated by TTX administration, as shown in Figure 1. The genes downregulated three-fold and more were extracted and are listed in Table 3. The highest downregulated gene in the TTX-administered group was elongation factor G2, and its FC value was −13.0. As shown in Figure 3, gene ontology classification of the downregulated genes showed that those involved in protein binding and enzyme and cofactors (metabolism) accounted for 20.8% and 13.1%, respectively, whereas those involved in the transcription factor, receptor activity and transporter activity accounted for 15.9, 10.1 and 6.6%, respectively.

**Figure 4:**
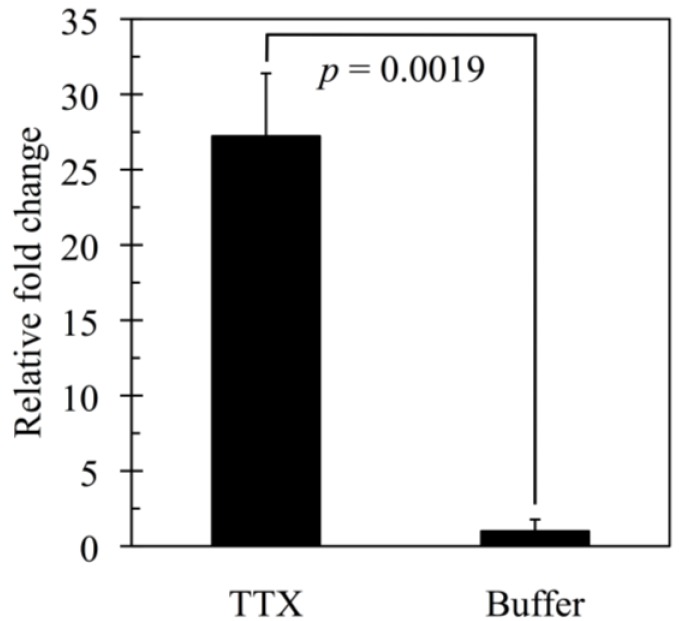
Real-Time PCR of the gene encoding chymotrypsin-like elastase family member 2A in the liver of *Takifugu rubripes* from both TTX- and buffer-administered groups. Each value represents the mean ± SE of three individuals, each performed in duplicate.

To confirm the validity of the microarray data, real-time PCR was performed for the highest upregulated gene, chymotrypsin-like elastase family member 2A (Figure 4). There was a significant difference in the mRNA expression level between the TTX-administered (27.22 ± 4.18) and control group (1.00 ± 0.77) (*p* = 0.0019). This result is well correlated with the data of microarray analysis, FC 37.6 (Table 2).

**Table 3:** List of genes downregulated in the liver of the TTX-administered pufferfish *Takifugu rubripes* group compared to those in the buffer-administered control group (FC ^1^ > 3.0).

Ensemble ID	Gene name	Predicted description		Functional classification		FC	
ENSTRUT00000047556	Fusa2	Elongation factor G 2		Transcription factor		−3.0	
ENSTRUT00000009260	Rspo3	R-spondin-3		Transduction		−7.8	
ENSTRUT00000029878	Ncoa2	Nuclear receptor coactivator 2		Transcription factor		−7.3	
ENSTRUT00000005082	Sasb	Fatty acyl-CoA hydrolase precursor, medium chain		Enzyme, cofactor		−6.2	
ENSTRUT00000042623	Kcnj3	G protein-activated inward rectifier potassium channel 1		Ion channel activity		−5.9	
ENSTRUT00000029012	Galnt1	Polypeptide N-acetylgalactosaminyltransferase 1		Enzyme, cofactor		−5.7	
ENSTRUT00000034755	Stk11ip	Serine/threonine kinase 11-interacting protein		Protein binding		−5.5	
ENSTRUT00000007314	Unc5d	Netrin receptor UNC5D		Receptor activity		−5.3	
ENSTRUT00000024559	Dnmt3a	DNA (cytosine-5)-methyltransferase 3A		Transcription factor		−4.7	
ENSTRUT00000028271	finTRIM	Fish virus induced TRIM protein		Metal ion binding		−4.7	
ENSTRUT00000021561	Synpo2	Synaptopodin-2		Protein binding		−4.4	
ENSTRUT00000035155	Klhl8	Kelch-like protein 8		Protein binding		−4.4	
ENSTRUT00000013283	Lnx2	Ligand of Numb protein X2		Metal ion binding		−4.3	
ENSTRUT00000005282	Sox5	*T. rubripes* transcription factor SOX5 (AY277973.1)		Transcription factor		−4.1	
ENSTRUT00000004950	Egr3	Early growth response protein 3		Transcription factor		−4.0	
ENSTRUT00000001876	Nme1	Nucleoside diphosphate kinase A		Transcription factor		−4.0	
ENSTRUT00000037748	Cadm2	Cell adhesion molecule 2		Protein binding		−3.9	
ENSTRUT00000029148	Cln3	Battenin		Enzyme, cofactor		−3.8	
ENSTRUT00000018412	Hecd3	Probable E3 ubiquitin-protein ligase HECTD3		Protein binding		−3.7	
ENSTRUT00000010231	Wipi2	WD repeat domain phosphoinositide-interacting protein 2		Enzyme, cofactor		−3.7	
ENSTRUT00000038056	Angpt2	Angiopoietin-2		Receptor activity		−3.7	
ENSTRUT00000011781	-	Putative F-type lectin		Sugar binding		−3.6	
ENSTRUT00000025271	Tyro3	Tyrosine-protein kinase receptor TYRO3		Transduction		−3.6	
ENSTRUT00000036834	Pcolce	*T. rubripes* procollagen C-endopeptidase enhancer 1 (AF016494.1)		Protein binding		−3.4	
ENSTRUT00000009581	Rims1	Regulating synaptic membrane exocytosis protein 1		Protein binding		−3.4	
ENSTRUT00000013940	Pdlim5	PDZ and LIM domain protein 5		Protein binding		−3.4	
ENSTRUT00000041778	Foxa3	*T. rubripes* folkhead transcription factor FoxA3 (AB604763.1)		Transcription factor		−3.3	
ENSTRUT00000026385	Fam70a	Protein FAM70A		Unknown		−3.3	
ENSTRUT00000003229	Arhgef26	Rho guanine nucleotide exchange factor 26		Transcription factor		−3.2	
ENSTRUT00000007982	Kif2c	Kinesin-like protein KIF2C		Protein binding		−3.2	
ENSTRUT00000013282	Lnx2	Ligand of Numb protein X2		Protein binding		−3.2	
ENSTRUT00000005850	Crybb2	Beta-crystallin A2		Protein binding		−3.2	
ENSTRUT00000003860	Edaradd	Ectodysplasin-A receptor-associated adapter protein		Protein binding		−3.1	
ENSTRUT00000015819	Pbxip1	Pre-B-cell leukemia transcription factor-interacting protein 1		Transcription factor		−3.1	
ENSTRUT00000011222	Serpinh1	Serpin H1		Protein binding		−3.1	
ENSTRUT00000020096	Atp2b3	Plasma membrane calcium-transporting ATPase 3		Transporter activity		−3.1
ENSTRUT00000009874	Plxdc1	Plexin domain-containing protein 1		Unknown		−3.1
ENSTRUT00000047512	Suox	Sulfite oxidase, mitochondrial		Enzyme, cofactor		−3.1
ENSTRUT00000020028	finTRIM	Fish virus induced TRIM protein		Metal ion binding		−3.0
ENSTRUT00000041535	Pxn	Paxillin		Protein binding		−3.0
ENSTRUT00000033313	Nox5	*T. rubripes* NADPH oxidase 5 (BR000279.1)		Enzyme, cofactor		−3.0
ENSTRUT00000027204	Pacs2	Phosphofurin acidic cluster sorting protein 2		Unknown		−3.0
ENSTRUT00000044222	Tgfbrap1	Transforming growth factor-beta receptor-associated protein 1		Protein binding		−3.0
ENSTRUT00000027017	Hnrnpk	Heterogeneous nuclear ribonucleoprotein K		Transcription factor		−3.0
ENSTRUT00000043644	Hsp90b1	Heat shock protein 90kDa beta member 1		Protein binding		−3.0
ENSTRUT00000043157	Tle3	*T. rubripes* transducin-like enhancer protein 3 (AB236415.1)		Transcription factor		−3.0
ENSTRUT00000022207	Vwa1	Von Willebrand factor A domain-containing protein 1		Unknown		−3.0
ENSTRUT00000026592	Tacr3	Neuromedin-K receptor		Receptor activity		−3.0
ENSTRUT00000028610	Dach1	Dachshund homolog 1		Protein binding		−3.0
ENSTRUT00000011629	Gas2l3	GAS2-like protein 3		Protein binding		−3.0
ENSTRUT00000018505	Epha2	Ephrin type-A receptor 4 precursor		Receptor activity		−3.0

^1^ FC is the average fold change of the TTX-administered (n = 5) compared to buffer-administered control group (n = 5).

## 4. Discussion

In this study, we performed a single intramuscular administration of TTX to cultured marine pufferfish specimens of *T. rubripes* and DNA microarray gene expression analysis on Day 5 after the administration to identify genes possibly related to TTX accumulation in the liver.

TTX was detected in the liver, skin and ovary, but not in the muscle and testis of the pufferfish specimens in the TTX-administered group. The amount of TTX was highest in the liver and skin. The skin accumulated 28 ± 5% of the administered dose at the same level as that of the liver (28 ± 6%) on Day 5. We previously reported that the liver and skin of cultured pufferfish specimens of *T. rubripes* (940–1120 g body weight) accumulated up to 63 ± 5% and 9 ± 3% of the administered dose of 0.25 mg TTX/kg body weight at 60 min after intravascular administration, respectively [17]. In this connection, the examination of the tissue distribution of ^3^H-labeled TTX in cultured *T. rubripes* (90 and 110 g body weight) revealed that the total radioactivity was distributed mainly in the skin (45.1% and 54.1%, respectively), muscle (7.4% and 8.0%, respectively) and liver (19.0% and 15.7%, respectively) on Day 6 after intraperitoneal administration of ^3^H-labeled TTX [34]. In addition, Honda *et al.* [5] performed the feeding experiments, in which zero-year- and one-year-old pufferfish specimens of cultured *T. rubripes* were reared for 60 days with various types of TTX-containing diets, and revealed that the test fish accumulated a small amount of TTX (less than 3 MU (mouse unit)/g in most cases) mainly in the skin and liver at low doses (0.1 MU/g body weight/day) and a large amount (up to 57 MU) mostly in the liver and ovary at higher doses (0.2–1.0 MU/g body weight/day). Moreover, Ikeda *et al.* [35] examined the transfer profile of intramuscularly administered 50 MU of TTX to the cultured young immature pufferfish *T. rubripes* (approximately four-months-old, 13.2 ± 3.4 g body weight). They reported that TTX tends to be transferred to the skin from the other tissues, such as the liver and circulating blood, and that the total amount of TTX remaining in the entire body at 72–168 h after administration was approximately 60%–80%. These results suggest that TTX was transferred to skin tissues regardless of the administration routes and would be released from the skin tissues to excrete excess TTX or as a biologic defense substance against predators [36,37,38].

Lee *et al.* [22] previously reported three fibrinogen-like protein genes expressed in toxic liver of two different pufferfish, akamefugu *T. chrysops* and kusafugu *T. niphobles*. However, the expressions of these genes were not observed in this study. Little is known about the timing of expression of these genes after the toxification of pufferfish liver. The other possibility is that these genes were tremendously expressed, and their transcripts were too highly labeled with Cy3 to be measured by microarray analysis. Further investigations are required about the relationship between the hepatic toxicity and the expression mechanism to understand the functions of these genes.

Matsumoto *et al.* [23] examined the hepatic gene expression profile of cultured *T. rubripes* at 12 h after intramuscular administration of TTX by suppression subtractive hybridization and found that upregulated genes encoded acute-phase response proteins, including hepcidin, complement components, serotransferrin, apolipoprotein A-1, high temperature adaptation protein Wap65-2, fibrinogen beta chain and 70 kDa heat-shock protein 4, in the liver. In this study, the increased expression of these genes were not detected, suggesting that these proteins subsided within five days after intramuscular administration of TTX.

Feroudj *et al.* [24] performed DNA microarray analysis with total RNAs from toxic and non-toxic wild pufferfish, demonstrating that 1108 transcripts were more than two-fold higher in toxic than nontoxic specimens. The expression levels of nine genes were upregulated more than 10-fold in toxic and proteins encoded by these genes were related to vitamin D metabolism and immunity.

Yotsu-Yamashita *et al.* [39] reported liver-specific expression of pufferfish saxitoxin and tetrodotoxin binding protein (PSTBP) in the marine pufferfish, *T. pardalis*. In addition, Tatsuno *et al.* [40] found four genes (Tr1–Tr4) encoding PSTBP homologs from the publicly available Fugu genome database and revealed the constitutive expression of two distinct isoforms (Tr1 and Tr3) in the liver of cultured non-toxic *T. rubripes* specimens, declining in their toxin-triggered gene expression. In this study, the expression change of genes encoding PSTBP homologs was hardly observed. PSTBP and its homologs may have functions to bind toxic substances other than TTX, when TTX is absent.

In the TTX-administered group, 59 and 427 genes were significantly upregulated and downregulated, respectively, in comparison with the buffer-administered control group (two-fold change, *p* < 0.05). The highest upregulated gene was chymotrypsin-like elastase family 2A (Cela2a), known as elastase-2A, with 37.6 FC. The validity of this value was confirmed by real-time PCR analysis, indicating a good quantitative performance of the microarray analysis. A homologous gene encoding elastase 2A was first cloned from the human pancreas [41]. Further details about human pancreatic elastase has recently been revealed through the human gene project, and the expression of human elastase 2A gene encoding “neutrophil elastase” has been found to be regulated by hematopoietic transcription factors, such as AML1, C/EBPα, PU.1 and c-Myb transcription factors [42,43]. However, there is still limited information on secretion in pancreatic juice [44]. Although *T. rubripes* Cela2a is estimated to correspond to elastase 2A in hepatopancreatic juice, which digests elastic and fibrous proteins, little information is available on the gene expression and regulation mechanism of *T. rubripes* Cela2a. One possibility is that the hepatopancreatic digestion is activated during enterohepatic metabolism of TTX in the pufferfish liver.

This study demonstrated the upregulation of the sodium channel beta-2 subunit gene (Scn2b, FC value of 4.0) by TTX administration. The sodium channel beta-2 subunit modulates the kinetics of channel gating, as well as the stabilization and location of TTX-sensitive voltage-gated sodium channels [45,46,47]. Pertin *et al.* [48] reported a marked upregulation of the beta-2 subunit in the spared nerve injury model of rat. Lopez-Santiago *et al.* [49] also reported that beta-2 subunit modulates mRNA and protein expression of TTX-sensitive voltage-gated sodium channels. These findings suggest that TTX accumulation in pufferfish liver affects the expression and composition of voltage-gated sodium channels.

Dysferlin gene (Dysf, an FC value of 4.1) was also upregulated in the liver of the TTX-administered group. Dysferlin is a ubiquitously expressed transmembrane protein involved in Ca^2+^-mediated plasma membrane repair, vesicle fusion and Ca^2+^ homeostasis in skeletal muscle, regulating cell adhesion in human monocytes [50,51,52]. Oulhen *et al.* [53] suggested that dysferlin is essential for endocytosis oogenesis and embryogenesis in the sea star, *Patiria miniata*. TTX accumulation may damage plasma membranes, and thus, the upregulation of dysferlin found in this study may be related to the upregulation of the sodium channel beta-2 subunit gene, because channel proteins fuse with the plasma membrane.

Kitamura *et al.* [54] investigated gene expression changes in the cerebral cortical cells from E18 rat embryos by DNA microarray analysis in the presence and absence of TTX. They identified genes involved in the postsynaptic scaffold, regulation of actin dynamics, synaptic vesicle exocytosis and regulation of G-protein signaling as those downregulated in the presence of TTX and upregulated in the absence of TTX. In the present study, Rho GTPase-activating protein 29 gene (Arhgap29) was upregulated with 12.1 FC on Day 5 after TTX administration. Arhgap29 is a negative regulator of the Rho GTPase signaling pathway, which controls cytoskeletal rearrangement in human and other organisms [55,56,57]. This study also demonstrated the upregulation of the probable G-protein-coupled receptor 22 gene (GPR22, 3.3 FC), the GTP-binding protein Rheb gene (Rheb, 3.2 FC), Arf GAP with GTPase domain ankyrin repeat and the PH domain 2 gene (Agap2, 3.0 FC). Recently, Adams *et al.* [58] have found that the GPR22 gene is selectively expressed in the brain and heart of human and rodents, suggesting a possible role of GPR22 protein in the regulation of cardiac contraction. However, natural ligands for this receptor remain to be understood, and its function in other animal tissues is also unclear. Rheb protein is a molecular switch in many cellular processes, such as cell volume increase, cell cycle progression, neuronal axon regeneration, autophagy regulation, nutritional deprivation, cellular stress resistance and cellular energy control [59,60]. On the other hand, genes encoding the G-protein-activated inward rectifier potassium channel 1 gene (Kcnj3, FC value of −5.9) and the Rho guanine nucleotide exchange factor 26 gene (Arhgef26, FC value of −3.2) were downregulated in the liver of the TTX-administered group. As is well known, these genes are related to a G-protein-coupled receptor signal transduction system, suggesting that receptors and signaling pathways involved in cellular response to TTX may exist in pufferfish liver to reduce the toxic effect and to accumulate TTX.

It was demonstrated in the present study that the intramuscular administration of TTX influences the hepatic gene expression involved in gene transcription, the signaling pathway via receptors and channels and metabolic pathways. It has been reported that genes related to immunity and acute-phase responses were found to be upregulated in cultured *T. rubripes* on the intraperitoneal injection of TTX by SSH [23], in wild *T. chrysops* and *T. niphobles* by RAP RT-PCR [22] and in wild *T. rubripes* microarray analysis [24]. It is noted that samples were taken on Day 5 after TTX administration in the present study, differing from those taken at 12 h after administration of TTX for SSH, although both investigations adopted cultured *T. rubripes*. It may be important to have a G-protein-related signaling pathway for shifting acute-phase to steady-state metabolic responses. Alternatively, health conditions of pufferfish may cause varied gene expression patterns relating accumulation of TTX. Further investigation is needed to understand the biological significance of TTX in pufferfish.
